# Fidaxomicin for the Treatment of Clostridium Difficile Infection in the Pediatric Population - Not Quite So Soon Yet

**DOI:** 10.4021/gr318e

**Published:** 2011-05-20

**Authors:** Tanya Daniels, Tsz-Yin So

**Affiliations:** a University of North Carolina at Chapel Hill, Chapel Hill, NC 27514, USA; b Department of Pharmacy, Moses H. Cone Hospital, Greensboro, NC 27401, USA

**Keywords:** Fidaxomicin, Pediatrics, *Clostridium difficile*, Pharmacokinetic, Pharmacodynamic

## Abstract

Fidaxomicin is a new narrow spectrum macrocyclic antibiotic. It inhibits bacterial RNA polymerase and eradicates *C difficile* with minimal effect on normal intestinal flora. The US FDA granted orphan drug designation for all formulations of fidaxomicin for the treatment of *C difficile* infections in pediatric patients on January 10, 2011. Fidaxomicin has bactericidal activity against *C difficile* with a prolonged post-antibiotic effect. Even though this medication has an orphan designation for pediatrics, all the available pharmacokinetic and pharmacodynamic data were in subjects ≥ 18 years old. The MIC_90_ for fidaxomicin against *C. difficile* varies from 0.0078 to 0.25 mcg/ml. Fidaxomicin is poorly absorbed. The highest peak plasma concentration in patients treated with fidaxomicin was 0.191 mcg/ml. Fecal levels of fidaxomicin after oral administration are extremely high. The average fecal concentrations in *C difficile* patients were 255.6 mcg/g, 441.7 mcg/g, and 1443.3 mcg/g in the 100, 200, and 400 mg/day groups, respectively. At a dose of 400 mg/day the average fecal concentration was 5700 times higher than the highest MIC_90_ of fidaxomicin against *C difficile*. In a phase III clinical trial fidaxomicin 200 mg twice daily was compared with vancomycin 125 mg four times per day orally for 10 days. Only two patients were 18 years old, and no patients younger than 18 years old participated in the study. The rates of clinical cure with fidaxomicin were noninferior to those with vancomycin. Patients who were infected with non-North American Pulsed Field type 1 strains had fewer recurrences in the fidaxomicin group than patients in the vancomycin group. Side effects were similar between both therapies. Most patients experienced mild gastro-intestinal symptoms. Fidaxomicin is a good therapeutic alternative to vancomycin and metronidazole, especially in patients with recurrence of *C difficile* infection. Patients rarely experience systemic side effects which improves compliance. The dose of fidaxomicin is expected to be 200 mg given orally twice a day for patients 16 years and older. At this point, Optimer Pharmaceuticals Inc has conducted clinical trials in adults only. Additional clinical trials in pediatric patients are needed before therapeutic recommendations can be made in this population.

## Introduction

*Clostridium difficile* is a gram-positive, anaerobic, spore-forming bacillus that is responsible for the development of antibiotic-associated diarrhea and pseudomembranous colitis. Occasionally, antibiotic therapy results in a disturbance of normal bacterial flora of the colon. This leads to colonization with *C difficile* which releases powerful toxins that cause mucosal inflammation and damage [1]. *C difficile* infection usually manifests as mild-to-moderate diarrhea accompanied by abdominal cramping, but some patients present with acute abdomen and life-threatening fulminant colitis [2]. *C difficile* colitis is one of the most common nosocomial infections. Approximately 20% of hospitalized patients acquire *C difficile* during hospitalization [3]. This infection affects more than 700,000 people, including pediatric patients, in the United States (US) yearly [4]. *C difficile* infections are a well-known cause of diarrhea in children. The number of *C difficile* cases in children older than 1 year old more than doubled between 1997 and 2006. Pediatric patients with *C difficile* infection were younger and more likely to be Caucasians and to have private insurance [5]. Most patients recover without specific therapy, but in some cases symptoms can be prolonged and debilitating. Vancomycin or metronidazole along with discontinuation of precipitating antibiotic is currently used to treat *C difficile* colitis [6]. While initial response to these medications is good, resistant strains of *C difficile* are emerging, and the rate of recurrence is quite high. Thirty percent of patients experience a recurrence of illness within 60 days [2].

Fidaxomicin (Dificid™^) is a first representative of a new class of narrow spectrum macrocyclic antibiotic. Fidaxomicin is developed by Optimer Pharmaceuticals Inc., San Diego, CA. The US Food & Drug Administration (FDA) granted orphan drug designation for all formulations of fidaxomicin for the treatment of C difficile infections in pediatric patients 16 years old and younger on January 10, 2011. The drug is currently under consideration for standard approval by the FDA. Approval is expected to be in May 2011 [7]. In this article we aim to provide a review on the pharmacokinetic and pharmacodynamic profiles, dosage and tolerability, clinical effectiveness and clinical application of fidaxomicin in the treatment of C difficile infection; and to evaluate if this medication is appropriate to be used in the pediatric population with the available evidence in the literature. A PubMed search was conducted using four major search terms “fidaxomicin”, “pediatrics”, “pharmacokinetics” and “pharmacodynamics”. When “pediatrics” was used as a search term with “fidaxomicin”, no article was found.^

## Pharmacodynamic Profile/Mechanism of Action

Fidaxomicin acts by inhibiting sigma-dependent transcription of bacterial RNA polymerase and selectively eradicating *C difficile* with minimal effect on normal intestinal flora. Fidaxomicin has bactericidal activity against *C difficile* with a prolonged (> 24 hours) post-antibiotic effect. It demonstrates faster killing and longer antibacterial effect after drug removal in comparison with vancomycin. Even though this medication has an orphan designation for pediatrics, all the available pharmacokinetic and pharmacodynamic data were in subjects ≥ 18 years old. The value of MIC_90_ for fidaxomicin against *C. difficile* varies from 0.0078 to 0.25 mcg/ml depending on the *in vitro* conditions. The value of the MIC_90_ increased 2 to 8 fold at more alkaline pH. Spontaneous resistance was rare. There has been no cross-resistance with azithromycin, ampicillin, telithromycin, ciprofloxacin, metronidazole, vancomycin, rifampin, and rifaximin. Fidaxomicin lacks activity (MIC > 16 mcg/ml) against Gram-negative anaerobes and facultative aerobes (i.e., *Bacteroides* species, enterobacteriaceae, *Haemophilus* species, and *Pseudomonas aeruginosa*). It demonstrates activity (MIC < 2 mcg/ml) against some Gram-positive anaerobes other than *C difficile* (i.e., *Peptostreptococcus* species, *Clostridium perfringens*, and some lactobacilli) but poor activity (MIC > 4 mcg/ml) against many other Gram-positives organisms, such as *Staphylococcus aureus* (both methicillin-susceptible and resistant) and *Enterococcus faecium* (both vancomycin-susceptible and resistant) [8].

## Pharmacokinetic Profile

Pharmacokinetics of fidaxomicin were investigated in healthy volunteers as well as in patients with *C difficile* infection who were ≥ 18 years old (49.3 ± 8.6 years). After multiple-dose administration of 100, 200, or 400 mg/day of oral fidaxomicin in both patients with *C difficile* infection and healthy adults, systemic absorption of fidaxomicin was low and plasma concentrations were mostly undetectable (< 5 mcg/ml). However, the proportion of patients with *C difficile* who had measurable plasma concentrations of fidaxomicin increased in a dose-dependent manner. The percentage of patients with measurable plasma fidaxomicin concentrations in the 100, 200, and 400 mg/day groups was 14.3, 56.3, and 81.3% respectively. The highest peak plasma concentration was 0.191 mcg/ml [9]. Fecal levels of fidaxomicin after oral administration are extremely high. In a phase II study, the average fecal concentrations in *C difficile* patients were 255.6 mcg/g, 441.7 mcg/g, and 1443.3 mcg/g in the 100, 200, and 400 mg/day groups, respectively. At a dose of 400 mg/day, the average fecal concentration was approximately 5700 times higher than the highest MIC_90_ of fidaxomicin against *C difficile* (0.25 mcg/ml). Fidaxomicin forms fecal metabolite, which is also highly active against *C difficile*. Urinary levels of fidaxomicin were below the limit of detection in all specimens obtained. Half-life of fidaxomicin is between 0.94 and 2.77 hours. Due to low systemic absorption, no adjustment is necessary for patients with renal and hepatic impairment. No drug interactions were reported [10]. (Table 1)

**Table 1:** Summary of Fidaxomicin’s Pharmacokinetic Data in Adults [9, 10]

Absorption	Very poor
Distribution	Plasma concentration low (< 5 mcg/ml) Fecal concentration very high (1443.3 mcg/g)
Metabolism	No systemic metabolism Forms fecal active metabolite
Excretion	Fecal Urine concentrations undetectable
Half Life	0.94 - 2.77 hours

## Dosage/Tolerability

In a phase II trial adult patients were taking 50 mg BID, 100 mg BID, or 200 mg BID of oral fidaxomicin. Subjects assigned to the 200 mg BID group experienced the highest rates of clinical cure. Fidaxomicin administered at this dose was very well tolerated. Nine out of forty five subjects (four patients in each of the lower dosage groups and one patient in the 400 mg/day group) reported adverse events. All adverse events (fall, shortness of breath, pain in the extremity, renal colic, bronchitis, pneumonia, urinary tract infection, hypotension, fluid overload, pancreatitis, diarrhea, cardiac failure, angina, cerebro-vascular damage, gastro-intestinal bleeding, and *Staphylococcus aureus* bacteremia) appeared not to be related to the study medication [11, 12].

## Phase III Clinical Trial

Phase III prospective, multicenter, double-blind, randomized, parallel-group, non-inferiority clinical trial compared the efficacy and safety of fidaxomicin with those of vancomycin for the treatment of *C difficile* infection. Six hundred twenty nine adult patients (mean age 61 years) were enrolled in the US and Canada. Subjects who were older than 16 years old could participate in the study; but at the end of the enrollment, only two patients were 18 years old, and no patients younger than 18 years old participated in the study. All patients had acute symptoms of *C difficile* infection (more than three unformed bowel movements in the 24-hour period before randomization) and a positive result on a stool toxin test obtained within 48 hours before randomization. Subjects could have received up to four doses of metronidazole or vancomycin in the 24-hour period before randomization. Patients with life-threatening or fulminant *C difficile* infection, toxic megacolon, previous exposure to fidaxomicin, a history of ulcerative colitis or Crohn’s disease, or more than one occurrence of *C difficile* infection within 3 months before the start of the study were excluded.

Patients were randomly assigned to receive fidaxomicin 200 mg twice daily or vancomycin 125 mg four times daily orally for 10 days. Patients in the fidaxomicin group received antibiotic every 12 hours with intervening matching doses of placebo. Subjects in the vancomycin group received medication every 6 hours. Both study medications and placebo were encapsulated to look the same. The primary end point was clinical cure, defined as resolution of symptoms and no need for further treatment of *C difficile* infection as of the second day after the end of the course of therapy. The secondary end points were recurrence of *C difficile* infection (diarrhea and a positive result on a stool toxin test within 4 weeks after treatment) and global cure (cure with no recurrence).

Five hundred forty eight patients (87.1%) were evaluated for the per-protocol analysis. The rates of clinical cure with fidaxomicin were noninferior to those with vancomycin in both the modified intention-to-treat analysis (88.2% with fidaxomicin and 85.8% with vancomycin) and the per-protocol analysis (92.1% with fidaxomicin and 89.8% with vancomycin). Patients in the fidaxomicin group had fewer recurrences of the infection than patients in the vancomycin group and, as a result, had higher global cure rate. This was true for the intention-to-treat analysis (15.4% with fidaxomicin and 25.3% with vancomycin, P = 0.005) as well as for the per-protocol analysis (13.3% with fidaxomicin and 24% with vancomycin, P = 0.004). The lower rate of recurrence was seen in patients with non-North American Pulsed Field type 1 strains only. The side effect profile was similar between the two therapies. Most patients experienced mild gastro-intestinal symptoms. No subject discontinued the medication as a result of intolerance or allergy. Ninety five point eight percent (95.8%) of patients in the fidaxomicin group and 96.1% of patients in the vancomycin group were compliant with therapy [13]. (Table 2)

**Table 2:** Results of Fidaxomicin Versus Vancomycin for *C Difficile* Infection [13]

	Intention to Treat		Per Protocol	
	Fidaxomicin	Vancomycin	Fidaxomicin	Vancomycin
Clinical Cure*	88.2% (253/287)	85.8% (265/309)	92.1% (244/265)	89.8% (254/283)
Recurrence**	15.4% (39/253)	25.3% (67/265)	13.3% (28/211)	24.0% (53/221)
Global Cure***	71.6% (214/287)	64.1% (198/309)	77.7% (206/265)	67.1% (190/283)

*Clinical cure = resolution of symptoms and no need for further treatment of *C difficile* infection as of the second day after the end of the course of therapy

**Recurrence = diarrhea and a positive result on a stool toxin test within 4 weeks after treatment.

***Global cure = cure with no recurrence.

## Clinical Application

Fidaxomicin is a promising new drug for the treatment of *C difficile colitis* which has many advantages over metronidazole and vancomycin. Because of its narrow antimicrobial spectrum, normal intestinal flora is not affected. Unlike metronidazole, fidaxomicin is poorly absorbed. This allows substantial quantities of the drug to reach the colon. Patients also rarely experience systemic side effects, which can help improve compliance. Substantial reduction in *C difficile* (non-North American Pulsed field type 1 strains) recurrences in comparison with vancomycin is a major advantage of fidaxomicin. Increased activity of fidaxomicin against non-North American Pulsed field type 1 strains of *C difficile* can be explained by the differences in the sigma-factors exhibited by different strains. Bactericidal activity of fidaxomicin depends on the presence of certain sigma-factors in the bacteria. Fidaxomicin is a bactericidal antibiotic with prolonged post-antibiotic effect. These qualities provide advantage over vancomycin, which is a bacteriostatic antibiotic without post-antibiotic activity [8].

Fidaxomicin is a good therapeutic alternative to vancomycin and metronidazole, especially in patients who experienced recurrence of *C difficile* infection. Fidaxomicin can potentially be used as a first line treatment in patients who previously had fecal cultures positive for non-North American Pulsed field type 1 *C difficile* strains. Even though all these pharmacokinetic and pharmacodynamic data are currently only in adults, researchers potentially can use this information to conduct safety clinical trial in pediatric patients in the future. As a matter of fact, Optimer Pharmaceutical Inc. is developing an oral suspension formulation for pediatric patients with *C difficile* infection as required by the FDA [14].

## Conclusion

*C difficile* colitis can present a major problem in children treated with broad spectrum antibiotics. At this point, Optimer Pharmaceuticals Inc. has only conducted clinical trials in adults. The phase III study did not include any pediatric patients and had only 2 patients who were 18 years old. Additional pharmacokinetic/pharmacodynamic clinical trials in pediatric patients are needed before therapeutic recommendations can be made in this population. Metronidazole and oral vancomycin still should be the mainstay of therapy for *C difficile* infection in pediatric patients at this time.
